# Causality and Cointegration Analysis between Macroeconomic Variables and the Bovespa

**DOI:** 10.1371/journal.pone.0089765

**Published:** 2014-02-28

**Authors:** Fabiano Mello da Silva, Daniel Arruda Coronel, Kelmara Mendes Vieira

**Affiliations:** Federal University of Santa Maria, Department of Business, Santa Maria, Rio Grande do Sul, Brazil; University of Warwick, United Kingdom

## Abstract

The aim of this study is to analyze the causality relationship among a set of macroeconomic variables, represented by the exchange rate, interest rate, inflation (CPI), industrial production index as a proxy for gross domestic product in relation to the index of the São Paulo Stock Exchange (Bovespa). The period of analysis corresponded to the months from January 1995 to December 2010, making a total of 192 observations for each variable. Johansen tests, through the statistics of the trace and of the maximum eigenvalue, indicated the existence of at least one cointegration vector. In the analysis of Granger (1988) causality tests via error correction, it was found that a short-term causality existed between the CPI and the Bovespa. Regarding the Granger (1988) long-term causality, the results indicated a long-term behaviour among the macroeconomic variables with the BOVESPA. The results of the long-term normalized vector for the Bovespa variable showed that most signals of the cointegration equation parameters are in accordance with what is suggested by the economic theory. In other words, there was a positive behaviour of the GDP and a negative behaviour of the inflation and of the exchange rate (expected to be a positive relationship) in relation to the Bovespa, with the exception of the Selic rate, which was not significant with that index. The variance of the Bovespa was explained by itself in over 90% at the twelth month, followed by the country risk, with less than 5%.

## Introduction

After a long period of instability, economic indicators adopted in Brazil resulted in a certain level of stability, mainly since the second half of the 1990s. These policies were based mainly on parameters advocated by financial institutions such as the World Bank and the International Monetary Fund (IMF). Several changes in both the macroeconomic scenarios (particularly with the introduction of the Real Plan and of some macroeconomic measures such as a regime of inflation goals, fiscal responsibility law and the reduction of the debt/Gross Domestic Product (GDP) ratio) and in the regulatory frameworks (approval of Annex IV through Resolution no. 1832 of the National Monetary Council), made the Brazilian stock market attractive to international investors.

These measures resulted in the improvement of the conditions necessary for sustainable economic growth and in a capital market more attractive to foreign investors, since Brazil began to be recommended by rating agencies, presenting circumstantial evidences sufficient to achieve the investment grade [15].

In this sense, [1] point out that in recent decades, the interaction between macroeconomic variables and the behavior of the stock market has been a subject of interest among academics and market analysts. They argue that stock prices are determined not only by financial indicators, but for some macroeconomic variables such as interest rates, exchange rate, inflation indexes and industrial production, representing the economic activity.

[7] considers that investigations aimed to corroborate or refute the presence of causality relationships between stock market indexes and macroeconomic variables can provide relevant and original evidences related to the operation of the integration of those markets, as well as to contribute to the understanding of their dynamic balance mechanisms. This is because the macroeconomic variables may be useful as a measure of the future performance of the asset if they have direct relationship to their rise or fall movement.

With this concern in mind, the main objective of this study was to analyze the causality relationship of a set of macroeconomic variables and the Brazilian stock market, represented here by the Bovespa. Specifically, it was sought to verify the existence of a long-term relationship between macroeconomic variables and the Bovespa, by means of cointegration tests, considering, for that, the period from January, 1995 to December, 2010. To identify how variations in the Bovespa, are transmitted to the variations of macroeconomic variables over time, the impulse response function was calculated, considering a period of 10 months. And finally, in order to analyze the error variance percentage due to each each endogenous variable along the prediction horizon, the analysis of variance decomposition was used.

This work is structured in three sections other than this introduction. In the second section, the methodological procedures are presented, while in the third, the obtained results are analyzed and discussed, and finally, in the last section, some concluding remarks are presented.

## Methodology

### Analytical model

To check whether the series are stationary, the Augmented Dickey-Fuller (ADF) stationarity test and the nonparametric [14] test will be used. The first considers the autoregressive models of an order greater than the unity, as shown by the expression (1), described by [3]: 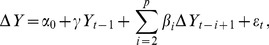
(1)


in which 
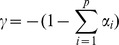
 and 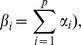
being that 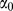 is the intercept ; 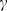, order of the autoregressive model which describes the behavior of the temporal series; Y, dependent variable; 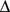
_,_ difference operator; and 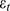, error structure, which is identically and independently distributed.

The parameter of interest in the regressions (without intercept and without trend, with only the intercept, with intercept and trend) is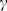, being that if 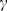 =  0, the series contains a unit root. In this test, the result of the *t* statistic is compared with appropriate values given by Dickey-Fuller to verify whether the null hypothesis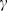 = 0 is accepted or rejected.

This hypothesis should be rejected when the calculated value of the *t* statistic exceeds the critical value of Dickey-Fuller, signaling that the series will be stationary; otherwise the series will not be stationary.

The Phillips-Perron (PP) test consists in a non-parametric alternative to the ADF test. This test can be used when the wastes are serially correlated.

In this case, the hypothesis employed in the Dickey-Fuller test that the error is identically distributed is disregarded. Moreover, the price series will not possess a unit root if the null hypothesis can be rejected; otherwise the series will be non-stationary [14].

Another analytical method employed in this study concerns the causality test suggested by [5]. This test verifies whether the incorporation of past values of an *X* variable contribute to better predictions for the *Y* variable. Thus, it is a test of temporal preceding and not of causality in the sense of a relation of cause and effect. This test requires the estimation of the following equations (2) and (3):
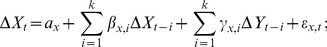
(2)

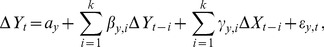
(3)


where: 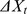
and 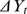 indicate the first difference of the variables 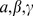 to be tested are the coefficients of the regressions to be estimated; 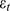 is the random error term.

The causality relationships between two variables can occur in the following ways:

i) Unilateral causality from 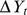 to 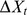: when the estimated coefficients in (2) for the lag variable 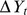 are jointly different from zero, and when the set of estimated coefficients in (3) for the variable 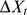 are not statistically different from zero; (ii) Unilateral causality from 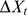 to 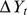: corresponds to the inverse of the previous form, that is, the null hypothesis is accepted in (2) and rejected in (3); (iii) bicausality or simultaneity: when the lagged coefficient sets of 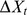 and 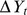 are statistically different from zero in both regressions; and (iv) absence of causality: is the contrary of (iii), that is, the null hypothesis is not rejected in (2) and (3).

According to [5], in a model with two variables, if there is a cointegration relationship between them, then there is causality in at least one direction. The econometric estimation of the price relationships considered in this work was based on the vectorial autoregression (VAR) model, whose representation of the VAR, of the *p* order, is expressed as follows according to Enders (1995):
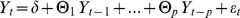
(4)


where each 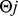 is a matrix of the *k* x *k*; parameters and 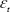 is a k-dimensional vector of white noise terms with covariance matrix 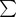.

The estimation of the lag order *p* of the VAR model will be obtained at the lowest *Akaike* (AIC), *Schwarz* (SC) and *Hannan-Quinn* (HQ) information criteria. According to [10], the coefficients of the equation (4) do not consider the relationship between the variables expressed in the VAR model. Therefore, the impacts of the innovations can be analyzed by impulse-response function, which provides the current and future effect on endogenous variables originating from a standard deviation of a shock in contemporary innovations, that is, outlines the behavior of the series included in the VAR model in response to shocks caused by residual variables.

Still in this perspective, [12] highlights another way to characterize the dynamic interrelationship between the variables of the model that can be captured by the decomposition of prediction errors variance for *k* periods forward. The decomposition of variance measures the relative contribution of each shock on the endogenous variables of the VAR system, that is, it is able to show the fraction of error variance designed for each value, which results from the effect of innovations themselves and those derived from innovations in another variable and evaluate the explanatory power of each variable at monthly intervals of time.

To verify the long-term relationship between the variables in this study, we opted for the Johansen cointegration method. According to [2], cointegration means that temporal non-stationary series and integrated ones of the same order share similar stochastic trends, that is, they present a balanced long-term relationship. [9] developed a cointegration methodology based on the position or rank (r) of the matrix 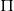, as presented in equation (5).
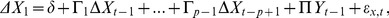
(5)


Determining the number of cointegration vectors requires knowledge about the position or rank (r) of the 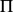 matrix. According to [2] there are three possibilities:

i) The position of 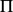 being complete. In this situation, any linear combination between the variables is stationary and the model adjustment shall be made with the variables in level; ii) the position of 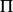 being null, so there is no cointegration relationship and the model must be adjusted with the variables in differences; iii ) the matrix 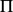 having a reduced position. In this case, there are *r* cointegration vectors, in which 0< r <n. and iv) [9] established two statistical tests aiming to discover the number of cointegrating relationships of the series 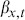. In this work, we used the trace tests and maximum eigenvalue test to identify the presence of cointegration vectors.

For [2], the trace test seeks to test the null hypothesis that the number of distinct cointegrating vectors is less than or equal to *r* (H_0_  =  cointegrating vectors 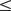 *r)* against the alternative hypothesis that the number of these vectors is greater than *r* (H_1_  =  cointegrating vectors>*r*) which can be expressed by (6):
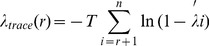
(6)


Where 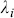 are the estimated values of the characteristic roots obtained from the 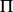 and T matrix, is the number of observations.

The maximum eigenvalue test aims to test the null hypothesis that the number of vectors is r (H_0_: cointegration vectors  =  r) against the alternative hypothesis of the existence of *r +1* cointegrating vectors (H_1_: cointegrating vectors  =  *r +1* ) which can be represented as follows (7): 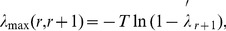
(7)


After detecting the cointegration relationship proposed by [4] between the variables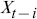 and 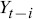 we passed on to the next step, which consists in the inclusion of the model of error correction, which has the advantage of retaining information about the level of the series, so that the long-term relationships between the variables of the studied model remain present. If the variables 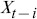 and 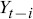 of the equation (5) are integrated from order 1 [I(1)] and there is a linear combination between them, which is an integrated from the order of zero [I (0)], we will have the following mode of error correction according to [4], as presented by Equation (8).
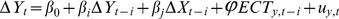
(8)

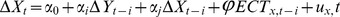



Where 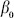, 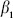, 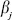, 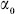, 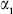 e 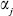 are the coefficients of the model; 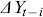 and 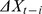 indicate the first difference of variables to be tested, lagged in *i* periods; 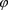 is the coefficient of long-term adjustment; 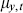 and 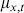 are the random error terms, and 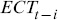 are the deviations from long-term balance between 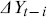 and 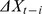, lagged in *i* periods given by the equation (5). If 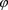 is statistically significant, the long-term cointegration equation errors are useful to adjust price variations in the short term, that is, we can verify which imbalance proportion in 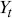 in one period is corrected in the next period.

### Data sources

We selected as relevant variables to analyze the nominal exchange rate, the country risk (EMBI+), the stock market (Bovespa index), the nominal short-term interest rates (Selic Over), inflation rate (IPCA) and Industrial Production Index (IPI) as a *proxy* for gross domestic product (GDP), being from the Institute of Applied Economic Research database [8] and in the Yahoo Finance database for the Bovespa series. All series were transformed in natural logarithm form seeking to smooth and normalize the deviations. The model is estimated in the econometric software Eviews 6.0. These series were taken, as aforesaid, for the period from January 1995 to January 2010. Moreover, it is also important to highlight that these variables were not deflated, because, according to [18] and [17] the deflation incorporates a trend in the data, so it does not make sense to include a trend prior to perform the cointegration test. Therefore, the series were measured in nominal form.

## Analysis and Discussion of Results

The test results converge indicating that, except for the inflation rate (IPCA), the other variables are not stationary in level, being integrated of the order equal to zero, I(0). Moreover, in first difference, they have indicated that there is no unit root, showing that the series are integrated of order 1, I(1). Based on these results the cointegration tests were estimated. Table 1 hereafter shows the tests results:

**Table 1:** ADF and PP tests results in level for the monthly series of the Bovespa, Selic rate, Exchange rate, PIB, IPCA and country risk logarithm series in January 1995 to December 2010.

	Level				First difference			
Variables	ADF^a^	Lags^b^	PP^c^	*τ crit* ^d^	ADF	Lags	PP	*τ crit*
Log (IBOV)	−2,659**	0	−2,824**	−2,876	−13,978**	0	−13,974**	−2,876
Log (IPCA)	−5,856*	0	−5,920*	−2,876	−13,203*	1	−23,496*	−2,876
Log (CAMBIO)	−2,049*	0	−2,051*	−2,876	−13,358*	0	−13,358*	−2,876
Log (SELIC)	−3,290**	1	−3,926**	−2,876	−18,169**	0	−18,183**	−2,876
Log (EMBI)	−1,514*	1	−1,332*	−2,876	−11,406*	0	−11,406*	−2,876
Log (IPI)	−0,797*	3	−0,592*	−2,876	−13,052*	0	−13,051*	−2,876

Source: Results obtained with the Eviews 6 software.

Note: a: Increased Dickey-Fuller test; b: Optimal value of selected lags according to the Schwarz criterion; c: Philips-Peron test; d: Critical values with 5% of significance; * model with only constant; ** model with Constant and trend.

Since the Johansen procedure is based on a VAR model, it is necessary to determine the number of lags of this model and to verify the presence or absence of deterministic terms to be included, being that they may be a constant, a trend or a Dummy variable.

In order to determine the number of lags (p) of the VAR model, three criteria were adopted: first, the decision by the number of lags (p) that minimized the Akaike Information Criterion (AIC), the second was the Schwarz (SC) criterion and, finally, the Hannan-Quinn (HQ) criterion. Table 2 present the results:

**Table 2:** Definition of the number of lags of the VAR model for the macroeconomic variables and for the Bovespa, January 1995 to December 2010.

Lags	LR	FPE	AIC	SC
0	-	8,25e-10	−3,888	−3,469
1	1648,394	9,38e-14	−12,971	−11,922*
2	136,253	6,18e-14	−13,390	−11,713
3	67,164	6,06e-14*	−13,414*	−11,107
4	43,342	6,84e-14	−13,300	−10,365
5	66,668*	6,56e-14	−13,353	−9,789

Source: Results obtained with the Eviews 6 software.

Note: * Indicates the lag order selected by the criterion; LR – Statistic of LR modifed sequential test; FPE – Final prediction error; AIC - Akaike Information criterion; SC – Schwarz Information criterion.

The Final Prediction Error (FPE) and Akaike (AIC) criteria indicated that the model must have three lags. In contrast, the Schwarz criterion (CS) recommends that the model should have only one lag and, finally, the Hannan-Quin information criterion (HQ) indicated that the most suitable number would be two. As the criteria indicated different number of lags, the choice was made based on the same number of lags indicated by most criteria. In this case, as two criteria (FPE and AIC) indicated three lags, this value was considered in other stages of the cointegration tests.

Once determined the number of lags of the VAR model, the test proposed by [9] was performed to verify the existence of long-term relationship between variables. The results obtained for the trace test presented in Table 3 show that the null hypothesis that the position of the cointegration matrix is null (*r* = 0) is rejected, at 5% of significance. Thus, there are at least two cointegration vectors establishing the relations of long-term balance between variables.

**Table 3:** Trace Test for cointegration of the series for the macroeconomic variables and for the Bovespa, January 1995 to December 2010.

Null	Alternative	Test	Critical
Hypothesis	Hypothesis	Statistics	value (5%)
r = 0	r>0	169,287	117,708
r≤1	r>1	96,049	88,803
r≤2	r>2	58,262	63,876
r≤3	r>3	33,471	42,915

Source: Results obtained with the *Eviews 6* software.

Note: the trace test indicates that there are two equations of cointegration.

The analysis of Table 4 indicates that the null hypothesis that there is at most one cointegration vector (r = 1) cannot be rejected at 5%. Therefore, the maximum eigenvalue test indicates that, at this level of significance, there is a cointegration vector.

**Table 4:** Maximum Eigenvalue Test for cointegration of the series for the macroeconomic variables and for the Bovespa, January 1995 to December 2010.

Null	Alternative	Test	Critical
Hypothesis	Hypothesis	Statistics	value (5%)
r = 0	r = 1	73,237	44,497
r = 1	r = 2	37,787	38,331
r = 2	r = 3	24,790	32,118
r = 3	r = 4	16,140	25,823

Source: Results obtained with the *Eviews 6* software.

Note: The Maximum Eigenvalue test indicates that there are two cointegrating equations.

Both tests indicated the rejection of the null hypothesis that there is no cointegration vector, being possible to state that the variables are cointegrated, that is, there is at least one long-term balance relationship between them. For the purposes of this study, we chose the number of cointegration equations defined by the Trace Test, which indicated two cointegration vectors statistically significant at the level of 5%.

Table 5 presents the first cointegration vector, normalized to the variable logarithm of the Bovespa index, which is the variable of interest in this work.

**Table 5:** Cointegration vector normalized to the logibov variable.

LogIbov	Const.	logIpi	logIpca	Logselic	Logcambio	Tend.
1	10,344	−4,496*	0,676*	−0,101	0,743*	−0,003
		(−7,436)	(7,434)	(−0,944)	(6,855)	(−0,196)

Source: Results obtained with the Eviews 6.0 software *Statistically significant at the level of 5%.

Note: Statistics in parenthesis refer to standard deviation of the estimated parameter. Const. Constant; Tend.: Trend.

It is important to note that the ordering of the variables was based on Granger’s block exogeneity test (*Block Causality Tests*), according to [2]. In this case, the variables are sorted based on the value of chi-square statistic, with most exogenous variables (lower statistics values) being placed before the more endogenous variables. The order of the variables was defined as follows: Bovespa (LogIbov), Country Risk (LogEmbi), Industrial Production Index (LogIpi), Inflation (LogIpca) Interest Rate (LogSelic) and exchange rate (LogCambio).

Based on the estimated cointegration vector, the ratio of long-term balance of the Bovespa index in relation to their determinants can be written and each of the *Xi* parameters as the elasticity of the Bovespa index to the macroeconomic variables can be interpreted. The reparameterized equation is defined as:
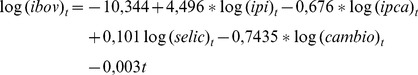
(9)


Based on these results, it can be said that most of the signals of the equation parameters (9) are in accordance with what was suggested by the economics theory.

The index of Industrial Production (logipi), used as a proxy of the GDP, had a positive and statistically significant value at the level of 5%. This result corroborates the result found by [16]. The same authors also point out that the growth in industrial production is a significant factor for the expansion of the stock market, represented here by the Bovespa. Inflation (logIPCA), in turn, presented a negative and significant relationship at the level of 5%. The negative response of stock prices for the best development of this economy is justified if the expected effects of a contractionary policy is higher than the expected gain due to increased production.

The Selic rate (logselic) presented a positive and insignificant parameter at the level of 5%. One possible explanation for this result, according to [13] is that the Central Bank does not consider the information contained in the Bovespa index variations in their decisions about the direction of interest rates. Finally, the exchange rate (logcambio) presented a negative and statistically significant parameter at the level of 5%. But a direct relationship with the Bovespa index was expected, because according to [11], there is a positive association between the currency devaluation and the rise of the Bovespa index.

The procedures performed to date have been useful to determine the long-term balance relationship between variables. However, [4] demonstrated that, even if there is a long-term balance relationship between non-stationary variables, it is possible for an imbalance to occur in the short term. Therefore, the VEC was estimated, using the VAR auxiliary model used for the cointegration test.

The long-term relationship between the variables, given by the cointegration vector and expressed in (4), was used as an explanatory variable of the error correction term. Table 6 shows which proportion of the short-term imbalance of the Bovespa index is corrected in the next period for the first and second cointegration vector.

**Table 6:** Coefficients of the VEC for the Bovespa index in relation to the other macroeconomic variables.

Variables	Coefficients	Standard error	T Statistic
ECT_1_	−0,078	0,037	−2,075*
ECT_2_	−0,060	0,023	−2,603*
LOGIBOV (−1)	−0,074	0,097	−0,764
LOGIBOV (−2)	−0,066	0,103	−0,644
LOGEMBI (−1)	0,015	0,076	0,208
LOGEMBI (−2)	0,005	0,070	0,083
LOGIPI (−1)	0,164	0,295	0,557
LOGIPI (−2)	0,173	0,273	0,634
LOGIPCA (−1)	0,070	0,035	2,012*
LOGIPCA (−2)	0,051	0,033	1,526
LOGSELIC (−1)	0,015	0,064	0,242
LOGSELIC (−2)	−0,060	0,064	−0,947
LOGCAMBIO (−1)	0,007	0,140	0,052
LOGCAMBIO (−2)	0,027	0,137	0,199
Dummy (CAMBIO)	0,112	0,045	2,468*
Dummy (ELEIÇÃO)	−0,054	0,036	−1,483
Dummy (CRISE08)	−0,022	0,040	−0,551
C	−0,064	0,035	−1,792

* Statistically significant at the level of 5%.

Source: Results obtained with the *Eviews* 6 software.

The estimation of the adjustment degree of the error correction terms (ECT), which measures the speed of convergence of short-term imbalance in relation to balance, verified that these terms were equal to −0.078 and −0.060, respectively, being both negative and significant at a level of 5%. In other words, the value of 0.078 provides that approximately 7.8% of the discrepancy between the actual value and the long-term or balance value are adjusted each month for the first vector and 6.0% for the second vector between the macroeconomic variables and the Bovespa. This result indicates that the first vector tends to fix the short term deviations more quickly in relation to the long-term balance.

The inflation rate, lagged in first difference, was the only one that presented a positive impact on Bovespa, at the significance level of 5%. This means that a variation of 1% in the IPCA variation in the preceding month will cause an increase of 0.7% in the Bovespa index. The other variables, lagged and in first difference, Bovespa, country risk, GDP, interest and exchange rate were not statistically significant in the short term, at the level of 5%.

**Figure 1:**
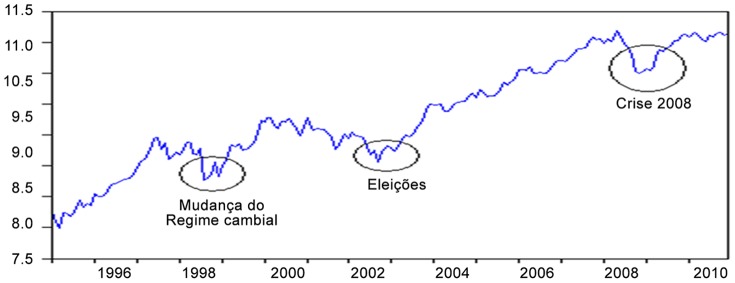
Description of dummy variables used in the period of January 1995 through December 2010. Source: Results obtained with the Eviews 6 software.

The finance literature emphasizes the influence of periods of financial crises and structural breaks in financial markets [17], so the effects of economic crises and possible structural breaks on the Bovespa were tested by the inclusion of dummy variables in the VEC model. In Figure 1 hereafter, the evolution of the BOVESPA (in logarithmic scale) is presented, highlighting the valleys affected by this index. These valleys are associated with economic events (highlighted in circles) that could have influenced the behavior of the BOVESPA.

The following dummy variables were used in the estimation of the results of this study: (a) change in the exchange rate regime (January 1999 onwards); (b) election of President Lula (April 2002 to November 2002) and (c) U.S. crisis of 2008 (October 2002 to April 2009). The dummy variable showed to be statistically significant only for the exchange rate, and its positive sign indicates that, during the change period of the exchange rate regime (January 1999 onwards), the standard was altered to levels above those observed before the change to the floating exchange rate regime. An increase of 1% when there was an exchange rate change caused an increase of 11.2% in the Bovespa index. The opposite sign was expected, since according to Pohlmann and Triches (2008), there was a drop in the stock market with the alteration of the exchange rate regime when the exchange rate became flexible, foreign investors saw the Brazilian economy with suspicion and there was a massive withdrawal of foreign capital in this period.

The existence of a cointegration relationship between the BOVESPA and the selected macroeconomic variables suggests that there must be at least one [5] causality direction between these variables. To determine the causality direction, we estimated the VEC that, apart from indicating the direction, allows to distinguish between short-term and long term causality. The causality test results via VEC are presented in Table 7.

**Table 7:** Causality test based on the VEC.

Dependent variable	Independent variable								
	Short term^a^							Short term^b^	
	IBOVESPA	EMBI	IPI	IPCA	SELIC	CAMBIO	TOTAL	ECT_1_	ECT_2_
IBOVESPA	-----	0,978	0,776	0,076***	0,545	0,980	0,525	−2,075**	−2,603**
EMBI	0,000**	-----	0,816	0,317	0,536	0,203	0,000	−0,268	0,854
IPI	0,035*	0,543	-----	0,166	0,003	0,708	0,001	4,107**	−1,198
IPCA	0,007**	0,162	0,002	-----	0,169	0,015	0,000	−3,661	3,878
SELIC	0,007**	0,865	0,000	0,193	-----	0,081	0,000	1,539	4,179
CAMBIO	0,538	0,017	0,070	0,123	0,017	-----	0,000	−5,858	−0,909

Source: Results obtained with the Eviews 6 software.

Note: ^a,b^ corresponds to the p-value of the *Wald Block Exogeneity* test and the *t* statistics of the term of error correlation, respectively. ** Significant at 1%, * significant at 5%, *** significant at a level of 10%.

Based on the results, it can be observed that in the short term, there was only one type of causality in a bidirectional manner between the IPCA and the Bovespa. In other words, any shock in the inflation affects the Bovespa in the short term and vice versa. The other variables (Country Risk, GDP, Selic and exchange rate) do not temporally precede, in the [5] sense, the Bovespa, because they did not reject the null hypothesis of the absence of causality. This result shows that any shock in one of these variables does not affect, in the short term, the other, in this case the Bovespa. The results presented in the same table indicated a unidirectional causality relationship between the Bovespa with the following variables: country risk, industrial production index and interest rate. The only variable that did not present statistically significant causality at 5% was the exchange rate.

Regarding the long term causal relationship between variables, it was found through the ECT coefficients (defined according to the Johansen trace test presented above) that they were significant at 1%. This means that there is a long-term causality between the error correction terms with the Bovespa. These results suggest that in the short-term the macroeconomic variables are adjusted to achieve their path of long-term balance.

Due to the difficulty of interpreting the estimated coefficients for the VAR model, it is common to summarize the results by means of the impulse-response function and of the variance decomposition. Due to the monthly frequency of the data, a 10-month period after the occurrence of the shocks is used for the analysis.

With regards to the impulse-response analysis, for the VEC (3), the trajectories of the Bovespa can be observed, not in terms of response to shocks in standard deviation, but in terms of elasticity in each of the studied macroeconomic variables. These estimates are presented as relative elasticities to initial unexpected shocks for all the variables given on the Bovespa index during ten months after that shock.

**Figure 2:**
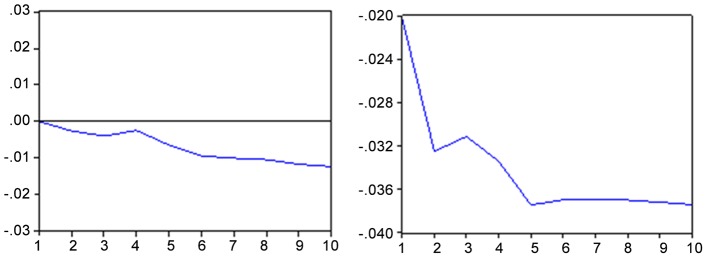
Impulse response function - IBOV x EMBI and vice versa. Response to the innovation of standard deviation Note: Period of 10 months Source: Results obtained with the Eviews 6 software.

The response of an unexpected shock in the Brazilian stock market causes a drop of about 0.37 percentage points in the exchange rate after five months (Figure 2 hereafter) remaining so until the end of the period (10 months). The negative impact of the variations in the exchange rate is more rapidly absorbed by the Bovespa index, reaching its maximum effect in the tenth month (about 0.11 percentage points).

**Figure 3:**
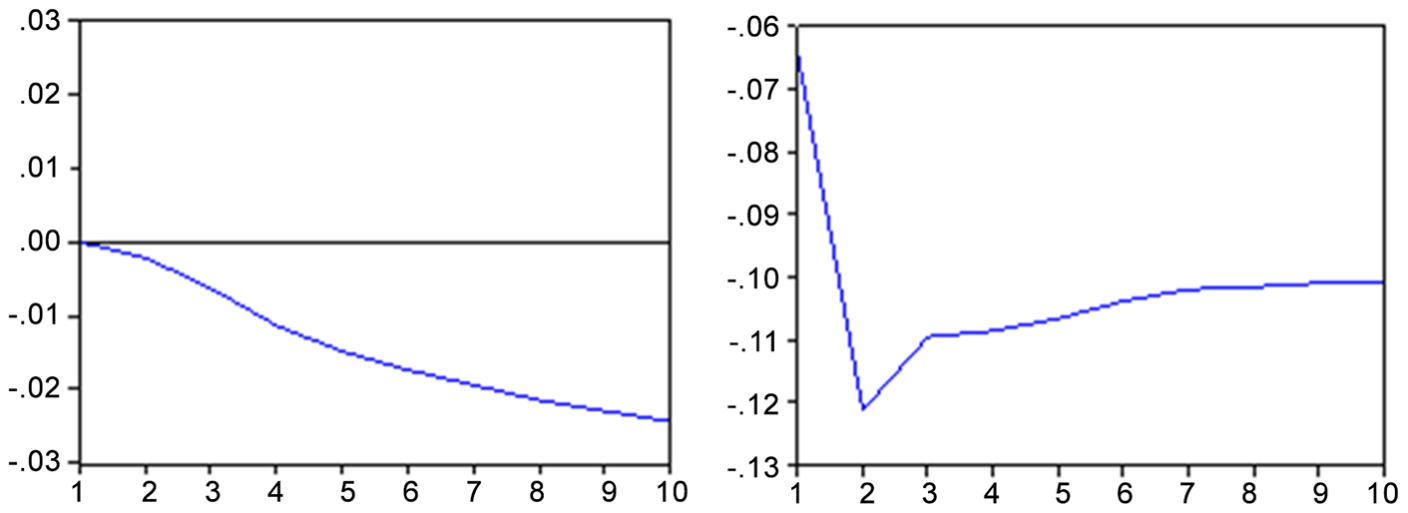
Impulse response function - IBOV x EMBI and vice versa. Response to the innovation of a standard deviation. Note: Period of 10 months. Source: Results obtained with the Eviews 6 software.

The response to an unexpected shock in the country risk causes a drop of about 0.20 percentage points in the Bovespa index (Figure 3 hereafter). In contrast, a shock in the Bovespa index causes a drop of 12 points in the second month and 11 points in the tenth month in the EMBI+. It is observed from this that the rating agencies have an important role as a “thermometer” of financial risk in emerging markets.

**Figure 4:**
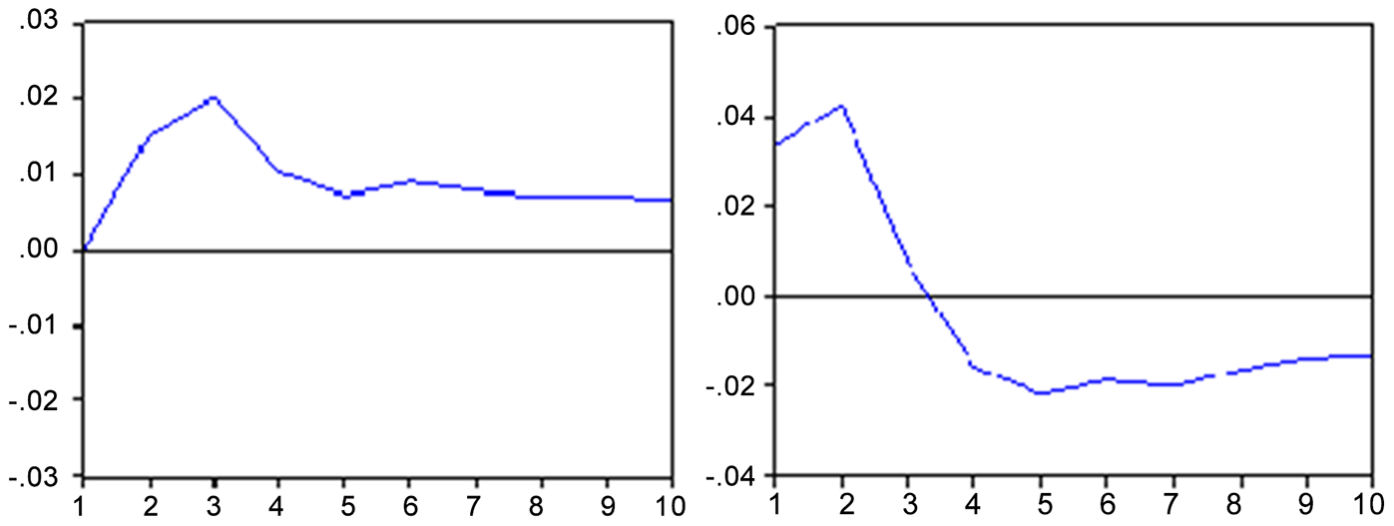
Impulse response function – IBOV x IPCA and vice versa. Response to the innovation of a standard deviation. Note: Period of 10 months. Source: Results obtained with the Eviews 6 software.

In the case of Bovespa’s innovation on the inflation rate, this can be proven by analyzing Figure 4 hereafter. A standard deviation shock causes a negative impact of approximately 0.30 percentage points over the inflation rate until the fifth month after the shock, having its effect gradually decreased but persisting during the ten months.

Moreover, an unexpected shock in the inflation rate has a moderated maximum positive effect on the Bovespa of about 0.30 percentage points at the third month, maintaining a small positive relationship in the ten subsequent months.

**Figure 5:**
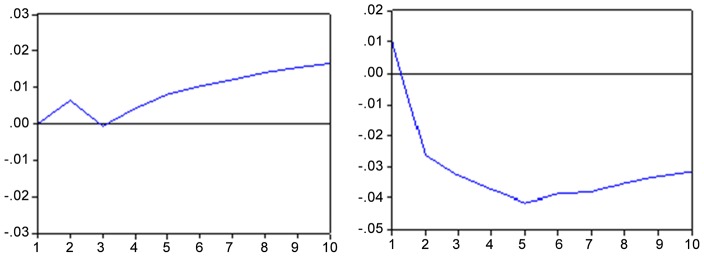
Impulse response function – IBOV x PIB (proxy IPI) and vice versa. Response to the innovation of a standard deviation. Note: Period of 10 months. Source: Results obtained with the Eviews 6 software.

Figure 5 hereafter shows that, when there is a shock in industrial production (IPI), the Bovespa index responds positively. On the other hand, for shocks of the real GDP, the answer of the Bovespa will be negative starting around the sixth month. Again, if the Bovespa reflects expectations about future events, it is likely that the stock market will not react positively to unexpected shocks on economic conditions.

**Figure 6:**
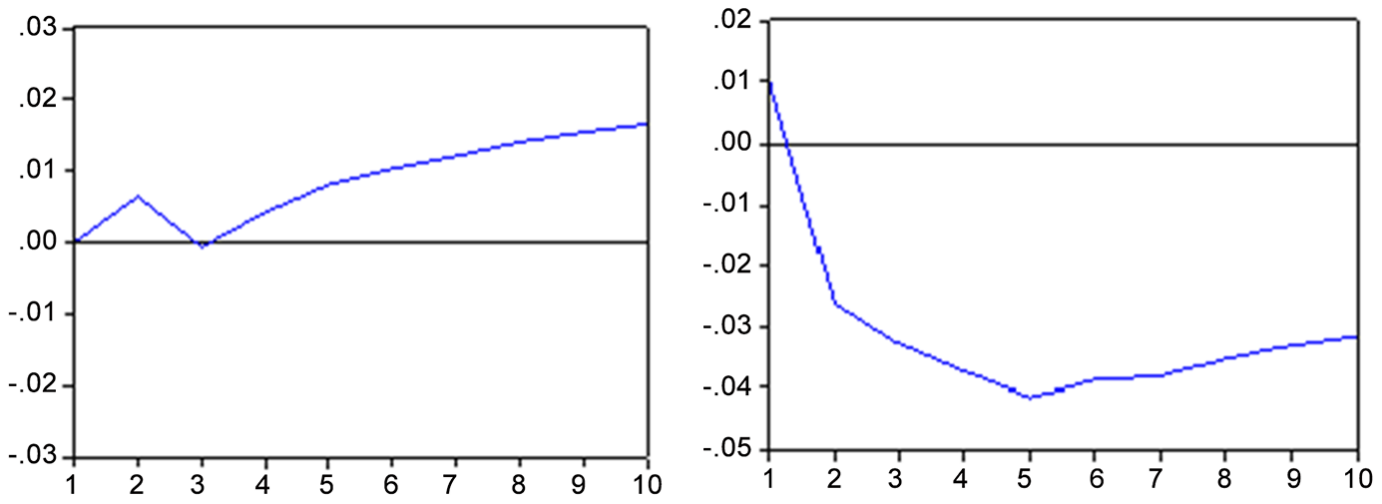
Impulse response function – IBOV x SELIC and vice versa. Response to the innovation of a standard deviation. Source: Results obtained with the Eviews 6 software. Note: Period of 10 months.

The response to an unexpected shock in the Brazilian stock market causes a drop of about 0.40 percentage points in interest rates after five months (Figure 6 hereafter) remaining so until the end of the period (10 months). In other words, it can be said that there would be a cash flow of the markets for debt securities to those of variable income, investments in stocks in the first ten months. Moreover, an unexpected shock in Bovespa leads to an increase of 0.10 percentage points after ten months. According to [6], a higher long-term interest rate will reduce the level of investment by firms (generating expectations of low return on assets).

Another useful tool in the VEC analysis is the decomposition of the variance, which allows us to say what percentage of the prediction error variance is due to each endogenous variable over the prediction horizon (Table 8).

**Table:** **Table 8**. Decomposition of the prediction errors variance of the log series (IBOV) log (IPCA) log (CAMBIO) log (SELIC) log (EMBI) log (IPI).

Accounted variables	Decomposition in the variable					
	Log (IBOV)		Log (IBOV)		Log (IBOV)	
	(%)		(%)		(%)	
Log (IBOV)	90,545	4,748	0,083	1,353	2,066	1,201
Log (IPCA)	3,696	3,814	34,726	50,060	6,977	0,724
Log (CAMBIO)	54,389	0,956	0,505	8,754	1,441	33,952
Log (SELIC)	15,792	26,926	4,431	8,132	41,717	2,999
Log (EMBI)	48,954	48,154	0,152	1,754	0,464	0,519
Log (IPI)	26,053	1,924	51,215	17,031	3,350	0,424

Source: Results obtained with the *Eviews 6* software. Note: Period of twelve months.

Analyzing the decomposition of the prediction errors variance, it can be observed that the Bovespa variance is explained in 90.54% in the twelfth month by itself. The second variable that presents the highest participation in the Bovespa shocks is the country risk, with approximately 5%, that is, the EMBI+ has a relatively small impact on the variance of the Bovespa. Regarding the inflation (IPCA) variance decomposition, it is observed that the same table demonstrated that 50.06% of the shocks on this variable is explained by itself and 34.72% by the Industrial Production Index (IPI). In turn, the unexpected shock in the exchange rate is explained in 54.38% in the twelfth month by the Bovespa, indicating that variations in the Bovespa may be important predictors of exchange rate. The unexpected shock in interest rates is explained in 41.71% in the twelfth month by itself, and approximately 27% of the variance of prediction errors is explained by the country risk.

By analyzing the variance decomposition of the penultimate variable (country risk) it was found that 48.95% of the shocks in this variable are explained by the Bovespa and 48.15% are explained by itself, indicating that perceptions of risk when the Brazilian economy would be represented largely by changes in the stock market. And finally, the Industrial Production Index variable as a proxy of the GDP is explained in 51.21% by itself and 26.05% by the Bovespa.

## Conclusion

The present study examined the existence of short and long term relationships among the selected macroeconomic variables, such as country risk, interest rate (Selic), exchange rate, industrial production index, inflation rate in relation to Bovespa. The [9] tests, through the statistics of the trace and of the maximum eigenvalue, revealed the existence of at least one cointegration vector. The results of the VEC estimates indicated that the lagged information represented by the macroeconomic variables, presented a short-term and long-term relationship with the Brazilian stock market. Regarding the inclusion of dummy variables in the model, the results indicated that the only dummy variable that showed a statistically significant relationship with the model was the exchange rate.

In the estimate of the first cointegration vector by the Johansen method, it was observed that the estimated parameters relating to inflation, GDP and exchange rate were statistically significant at the level of 5%. A positive relationship of the GDP with the Bovespa has been found. Now, the inflation and exchange rate presented a negative association with the Bovespa index. The interest rate (Selic) presented no long-term relationship with the Bovespa. In the [5] causality tests (1988) analysis, via error correction, it was found that there was a short-term causality only in a bi-directional manner between the IPCA and the Bovespa. The other variables (Country Risk, GDP, exchange rate and Selic) do not influence, in the Granger sense, the Bovespa, because they did not reject the null hypothesis of absence of causality. The presented results also indicated a unidirectional causal relationship between the Bovespa with the following variables: country risk, industrial production index and interest rate. The only variable that showed no causality was the exchange rate.

Regarding the long-term causal relationship between variables, it was verified through the ECT coefficients that they were significant at 1%. This means that the BOVESPA responds to long-term imbalances caused by macroeconomic variables.

In the variance decomposition analysis of prediction errors in VAR systems, it was found that the errors estimated in the twelfth month are explained in 90.54% by itself, although other variables, such as country risk, Selic rate, inflation, exchange rate and GDP have not shown a significant participation in the decomposition of prediction error variance of the Bovespa.

A limitation of this study is that the results, the analysis and discussions performed so far are only valid for the period in question, that is for the months of January 1995 through December 2010, because, as stated above, there are empirical studies that were conducted in different contexts (period of analysis and specificity of each country) who found no long-term relationship and/or of causality between macroeconomic variables and the stock market index.

For future studies, we suggest the extension of the present study to the stock markets of the major stock exchanges (Japan, USA etc....), to understand the effects of causality and motion direction with the Bovespa. As the Brazilian stock market is increasingly integrated into the process of financial globalization, any information on the international market, especially relative to stock indexes, could also influence the operations of buying and selling shares in the Brazilian market. Therefore, it would be interesting for other researches to verify whether such financial information have a degree of adjustment to more instantaneous and accurate to the stock prices than macroeconomic information.
